# Parastomal Evisceration: A Report of Two Cases and Review of Literature

**DOI:** 10.7759/cureus.5750

**Published:** 2019-09-25

**Authors:** Aditya A Kulkarni, Vivek Chauhan, Vishal Sharma, Harjeet Singh

**Affiliations:** 1 Surgery, Postgraduate Institute of Medical Education and Research (PGIMER), Chandigarh, IND; 2 Internal Medicine, Postgraduate Institute of Medical Education and Research (PGIMER), Chandigarh, IND

**Keywords:** ileostomy, colostomy, hernia, evisceration, complication, emergency, surgery

## Abstract

Intestinal stomas have been performed for hundreds of years for both benign and malignant disorders of the large and small bowel. Complications of stomas like stomal prolapse, parastomal hernia, and retraction are well-known. The evisceration of intra-abdominal contents is a very rare occurrence, carrying a high burden of morbidity. The etiology, timing, and treatment of this complication are not adequately described in the literature. We report two patients who were operated and in whom ostomy was fashioned; parastomal evisceration occurred in the early postoperative period in both cases. Both of these patients were operated emergently, and reduction of eviscerated contents followed by stoma refashioning was performed. One patient survived; whereas, the other patient who presented with septic shock expired due to multiorgan dysfunction syndrome. This report describes a rare and possibly fatal complication of ostomy and highlights the importance of meticulous operative technique in ensuring safety in this procedure.

## Introduction

Stoma formation, either temporary or permanent, is a commonly performed procedure in intestinal surgery. The creation of stoma carries major psychological sequelae for a patient. The stoma alters significantly the quality of life and the lifestyle of the patient. The performance of a stoma by itself is often regarded as a minor surgical procedure. However complications are frequent, especially in the early postoperative period [1]. Evisceration of intra-abdominal contents through the stoma site is one such rarely seen, devastating complication requiring urgent intervention. We report two such cases who presented to our surgical unit; we present the factors predisposing to intestinal evisceration and the therapeutic option as well as discuss their management. Our aim is to highlight a rare and possibly fatal complication of ostomy and underline the importance of meticulous operative technique in ensuring safety in this procedure. The consequences of improper stoma formation may be disastrous and surgical trainees should be adequately supervised and trained in this procedure, as a significant proportion of complications can be prevented by careful adherence to good surgical technique.

## Case presentation

Case 1

**Figure 1:**
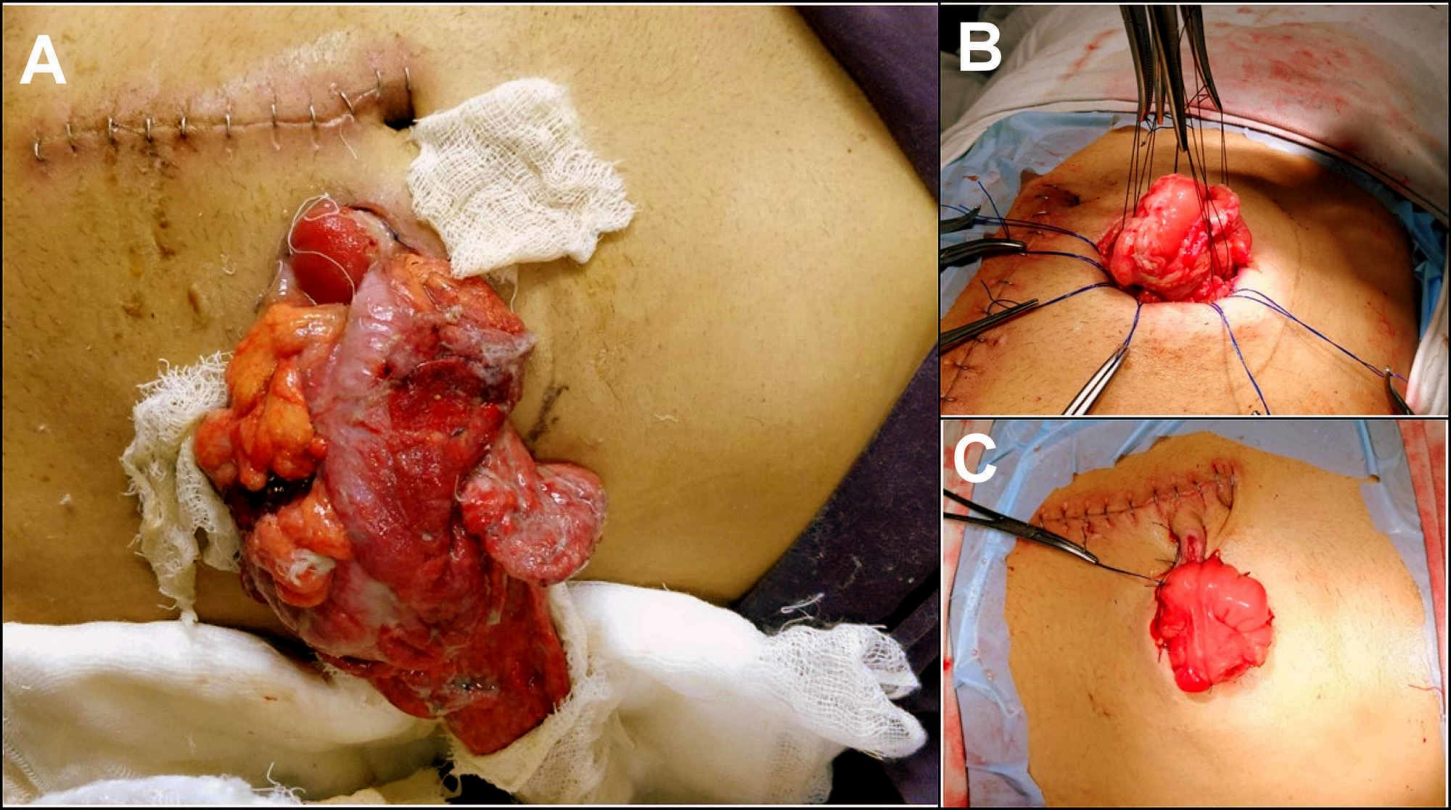
Operative picture of patient #1 A. Photograph showing the loop colostomy with transverse colon and omentum eviscerating outside. B. Intra-operative image showing the stoma being refashioned with nonabsorbable sutures taken to narrow the defect in the sheath. C. Final appearance of the refashioned stoma.

A 45-year-old male patient was operated for sacral chordoma and had an iatrogenic rectal injury during the surgery. The patient underwent repair of rectal injury and diverting loop sigmoid colostomy. The postoperative period was uneventful and he was discharged after nine days. Three days after discharge, he presented to the emergency department with severe pain and swelling at the colostomy site. This was preceded by a severe bout of coughing. On examination, a part of the large bowel and omentum was found to have eviscerated alongside the sigmoid colostomy through the same opening. The patient was taken for emergency surgery. The transverse colon and greater omentum were herniating from the medial aspect of the defect in the sheath at the colostomy site (Figure 1A). The sigmoid colon was found to be pushed laterally and was slightly retracted. The herniated contents were healthy and viable. The contents were reduced and the enlarged sheath opening was approximated on the medial aspect with nonabsorbable suture, to ensure a snug fit for the colostomy (Figure 1B). The skin opening was closed similarly, and mucocutaneous sutures were taken to mature the colostomy (Figure 1C). The patient did well postoperatively and was discharged on the fourth postoperative day.

Case 2

A 50-year-old female presented with perianal pain and induration. On evaluation, she was diagnosed with rectovaginal fistula secondary to locally advanced carcinoma cervix. Loop sigmoid colostomy was fashioned via left lower quadrant incision and brought out through the same incision. The stoma was matured over a tuberculin syringe hub as a temporary spur. The stoma functioned on day two and the patient was discharged on day three following surgery. Nine days following the first surgery, the patient presented to the emergency department with protrusion of bowel loops through the colostomy site. On evaluation, the patient was in sepsis with multiorgan dysfunction. After resuscitation, the patient was taken up for surgery. There was an evisceration of small bowel loops from the medial aspect of the colostomy site, and were contaminated with feces and were edematous. The previous incision was extended, the bowel loops were reduced after giving thorough lavage and the abdomen was closed. The stoma was refashioned. Postoperatively, she remained intubated and on vasopressors, with multiorgan dysfunction syndrome. She succumbed to her illness on day five.

## Discussion

Intestinal stoma creation is a commonly performed procedure as a part of operations performed for benign and malignant disorders of the small and large bowel. Common complications associated with stomas include skin excoriation, ischemia, stenosis, stomal retraction, stomal prolapse, and parastomal hernia [2]. However, parastomal evisceration of intraabdominal content (bowel, omentum) is an extremely rare complication with very few cases reported so far.

This devastating complication may present at any time after the initial surgery in which the stoma was created. In the majority of reported cases, evisceration was reported within a few days after the index surgery. However, this complication has been reported even one year following stoma creation [3]. In the immediate postoperative period, evisceration occurs due to mucocutaneous suture line disruption and as a result of ostomy wall necrosis in cases that present later on [4]. Both of our cases presented with the complication in the early postoperative period.

A search of the English literature on Pubmed and Medline revealed only 11 previously reported cases of parastomal evisceration (Table 1).

**Table 1:** Summary of cases of parastomal evisceration published in the English literature

Author, year	Age/ sex	Type of stoma	Time from surgery to evisceration	Predisposing factors	Management
Yucel ^[3]^, 2014	62/M	End descending colostomy	1 year	Chronic obstructive pulmonary disease (COPD), parastomal hernia increased intra-abdominal pressure	Reduction of eviscerated bowel and refashioning of stoma
Lolis ^[4]^, 2015	48/F	Transverse colostomy	18 months	Cough in postoperative period, chemotherapy, systemic corticosteroid use	Reduction of eviscerated bowel, colonic resection and refashioning of stoma
Ramly ^[5]^, 2013	81/M	End ileostomy	9 days	COPD, systemic corticosteroid use, poor nutrition, advanced age, ileostomy prolapse	Resection of nonviable bowel and creation of new ileostomy
Salles ^[6]^, 2011	62/M	Loop transverse colostomy	4 days	COPD, cough in postoperative period, increased intra-abdominal pressure, emergency surgery	Partial resection of transverse colon with terminal colostomy and mucosa fistula
Azouz ^[7]^,2014	69/M	End sigmoid colostomy	3 days	COPD, alcohol abuse, chronic smoker	Resection of nonviable bowel and refashioning of stoma
Villa ^[8]^, 2011	69/M	Loop transverse colostomy	8 months	Vomiting and retching, prior chemotherapy, increased intra-abdominal pressure, emergency surgery, ileostomy prolapse	Reduction of eviscerated bowel and refashioning of stoma
Arbra ^[9]^, 2017	90/M	End ileostomy	7 days	Advanced age, mechanical ventilation, emergency surgery	Reduction of eviscerated bowel and refashioning of stoma
Park ^[10]^, 2009	58/M	Temporary loop ileostomy	4 months	Chemotherapy, ileostomy prolapse	Resection of nonviable bowel and closure of ileostomy
Moffett ^[11]^, 2011	23/M	Ileostomy	Not reported	Increased intra-abdominal pressure	Reduction of eviscerated bowel and refashioning of stoma
Fitzgerald ^[12]^, 2008	65/M	Temporary loop ileostomy	10 days	None	Reduction of eviscerated bowel and refashioning of stoma

Due to the extreme rarity of this entity, it is difficult to pinpoint the factors predisposing to evisceration. However, a number of predisposing factors were identified in the previously reported cases like cough in the postoperative period, chronic obstructive pulmonary disease, increased intra-abdominal pressure, emergency surgery, use of corticosteroids, ileostomy/ colostomy prolapse, parastomal hernia, malignant colorectal neoplas­tic disease, and smoking and alcohol abuse. Creation of a larger stomal aperture to accommodate dilated bowel loop is also a possible cause, as reported by Salles et al. [6]. This was similar to what we encountered in our first patient, wherein the sigmoid colon was grossly distended and loaded with hard stool.

Treatment of evisceration depends upon the viability of the eviscerated bowel. Emergent repair is necessary as ischemia and infarction of bowel can be avoided with early surgical intervention. Eviscerated bowel should be reduced after enlargement of the hernia orifice, and nonviable bowel may need resection. The ostomy may be resited or refashioned with a reduction in fascial aperture.

## Conclusions

Stoma creation, though a relatively straightforward procedure, may be associated with significant complications like parastomal evisceration of intra-abdominal contents. Our case report highlights the importance of meticulous surgical technique in stoma creation and emergent surgical intervention when this rare complication occurs.
